# Novel global effector mining from the transcriptome of early life stages of the soybean cyst nematode *Heterodera glycines*

**DOI:** 10.1038/s41598-018-20536-5

**Published:** 2018-02-06

**Authors:** Michael Gardner, Andi Dhroso, Nathan Johnson, Eric L. Davis, Thomas J. Baum, Dmitry Korkin, Melissa G. Mitchum

**Affiliations:** 1 Division of Plant Sciences and Bond Life Sciences Center, University of Missouri, Columbia, USA; 2 Department of Computer Science and Bioinformatics and Computational Biology Program, Worcester Polytechnic Institute, Worcester, USA; 3 Department of Entomology and Plant Pathology, North Carolina State University, Raleigh, USA; 4 Department of Plant Pathology and Microbiology, Iowa State University, Ames, USA

## Abstract

Soybean cyst nematode (SCN) *Heterodera glycines* is an obligate parasite that relies on the secretion of effector proteins to manipulate host cellular processes that favor the formation of a feeding site within host roots to ensure its survival. The sequence complexity and co-evolutionary forces acting upon these effectors remain unknown. Here we generated a *de novo* transcriptome assembly representing the early life stages of SCN in both a compatible and an incompatible host interaction to facilitate global effector mining efforts in the absence of an available annotated SCN genome. We then employed a dual effector prediction strategy coupling a newly developed nematode effector prediction tool, N-Preffector, with a traditional secreted protein prediction pipeline to uncover a suite of novel effector candidates. Our analysis distinguished between effectors that co-evolve with the host genotype and those conserved by the pathogen to maintain a core function in parasitism and demonstrated that alternative splicing is one mechanism used to diversify the effector pool. In addition, we confirmed the presence of viral and microbial inhabitants with molecular sequence information. This transcriptome represents the most comprehensive whole-nematode sequence currently available for SCN and can be used as a tool for annotation of expected genome assemblies.

## Introduction

The soybean cyst nematode (SCN) *Heterodera glycines* is the most economically important pathogen of soybean, causing over one billion dollars in yield loss annually^1^. This microscopic roundworm begins its life cycle as an egg in the soil, undergoing one molt before hatching as a second-stage juvenile (J2). Once the nematode has hatched, it migrates through the soil towards a host plant where it invades the root tissue and migrates towards the vasculature, selecting a single cell to establish a feeding site called a syncytium. At this point, the nematode penetrates the cell wall using its stylet and releases a set of secretions into the host cell, including effector proteins. Stylet-secreted effector proteins identified to date share many characteristics including the presence of a signal peptide, lack of a transmembrane domain, and expression in the esophageal gland cells^2^. These effector proteins manipulate the host cell by modulating a variety of cellular processes to make it more suitable for the nematode, including suppression of host defense and stress responses and causing significant transcriptional re-programming in the host cell nucleus^3^. Effectors harboring nuclear localization signals are recognized by host cellular machinery for targeting to the nucleus where they modulate host nuclear functions^4^. Similar to effectors delivered by the stylet of piercing/sucking insects, the type III secretion system of bacteria or the haustorium of pathogenic fungi and parasitic plants, these effectors represent an interface between the nematode pathogen and host^5,6^. Once the feeding site is established, the nematode becomes sedentary and relies entirely on the host for nutrition for the remainder of its life cycle. The nematode slowly swells up as it undergoes a series of molts and differentiates into either a male or a female. Females protrude from the roots while the males regain mobility and exit the root to fertilize females, following which the males die. The females eventually die after fertilization, their bodies hardening into a protective casing for the eggs called a cyst that breaks off into the soil and begins the process anew. The early stages of the nematode infection cycle represent a key point in determining the fate of a cyst nematode. Whether or not the nematode will survive long enough to complete its life cycle depends on the ability of the nematode to survive and circumvent the hostile environment presented by the plant host.

In recent years, next generation sequencing technologies have been applied to several plant-parasitic nematode species, resulting in the assembly of complete genomes for *Meloidogyne hapla*, *M. incognita*, *Globodera ellingtonia, G.pallida*, *G. rostochiensis*, *Ditylenchus destructor*, and *Bursaphelenchus xylophilus*^7–12^. Despite the enormous economic importance of *H. glycines*, no finished and comprehensively annotated genome is currently available. In the absence of a sequenced genome, several other plant-parasitic nematode systems have turned to *de novo* transcriptome-level studies instead^13–16^. These studies were able to identify key features of the interaction of plant host and nematode pathogen, including the discovery of new effectors.

In the SCN system, studies have primarily focused on identifying and characterizing stylet-secreted effectors produced in the esophageal gland cells, which has resulted in the identification of 72 SCN effectors^17–19^. These studies based their identification of SCN effectors on the presence of a signal peptide as well as expression in the esophageal gland cells confirmed by in situ hybridization. Multiple functional studies have since been performed using these effectors, identifying host targets and characterizing their role in cyst nematode parasitism reviewed in^20^. Though the approach focused on the gland cells has been highly successful in identifying stylet-secreted effector proteins, low abundance transcripts, those harboring non-canonical secretion signals, and those encoding secreted proteins originating in other structures of the nematode, such as amphids^21^, are lacking. A global analysis allows for a comprehensive assessment of effectors, enabling studies to assess effector variation within and across populations to identify highly variable effectors potentially correlated with virulence, as well as those effectors highly conserved across the population that may be key components of the SCN infection process. Effector variation has been shown to be important in other plant pathogens such as bacteria and fungi as a tactic for evading host recognition and resistance^22,23^.

To provide comprehensive biological insight and a tool for comparative analyses between different nematode species and populations of *H. glycines* in the absence of a reference genome, a *de novo* transcriptome assembly of early life stages was generated. An analysis of the transcriptome confirmed previous reports of microorganisms present within the nematode with molecular details and identified new parallels to other plant-parasitic nematode species. We then performed multiple analyses focused on effectors; both predicting novel effectors using a newly developed bioinformatics tool called N-Preffector that is not reliant on the presence of a signal peptide and investigating variation of previously identified stylet-secreted effector protein sequences. This allowed for the identification of an additional suite of novel effectors that may play a pivotal role in SCN infection and could serve as potential targets for future development of novel SCN control strategies.

## Results

### Transcriptome sequencing and assembly

To gain global insights into the transcriptomic response associated with the establishment of SCN infection, mRNA sequencing of pre-parasitic second-stage juvenile (ppJ2) and parasitic second-stage juvenile (pJ2) life stages infecting a resistant and susceptible host was conducted, yielding a total of 603.6 million paired 100 base reads. Following initial filtering steps and removal of reads mapping to the soybean genome, the final input for transcriptome assembly was 430 million reads. Trinity *de novo* transcriptome assembly resulted in a final assembly of 147,910 transcripts with a total assembly length of 46.7 Mb and estimated 23-fold transcriptome coverage. The average length of these transcripts was 658 base pairs (bp) with a N50 of 1,085 bp (Table 1). When translated, 78,625 resulting proteins were predicted. This transcriptome assembly was then assessed using BUSCO (Benchmarking Universal Single-Copy Orthologs)^24^. Based on the 429 single copy orthologs for eukaryotes, the SCN assembly is 68% complete, with an additional 14% of the orthologs represented in fragmented transcripts and the remaining 18% missing from the transcriptome.

**Table 1:** *de novo* transcriptome assembly statistics for the SCN early life stage assembly.

Number of transcripts	147,910
Total assembly length (Mb)	46.7
Number of trinity ‘genes'	71,093
N50 (bp)	1,085
Maximum contig size (bp)	11,112 bp
Minimum contig size (bp)	201 bp
Average contig length (bp)	658 bp
Predicted proteins	78,625
BUSCO score	C: 68%, F: 14%, M: 18%

The assembly was generated from *H. glycines* pre-parasitic second-stage juvenile samples as well as parasitic second-stage juvenile samples from susceptible and resistant host interactions using the Trinity *de novo* transcriptome assembly tool. The transcriptome was assessed for completeness using the tool BUSCO (benchmarking universal single-copy orthologs) to identify complete (C), fragmented (F), and missing (M) sequences representing conserved orthologs found in all eukaryotes.

### Annotation of transcripts

**Figure 1:**
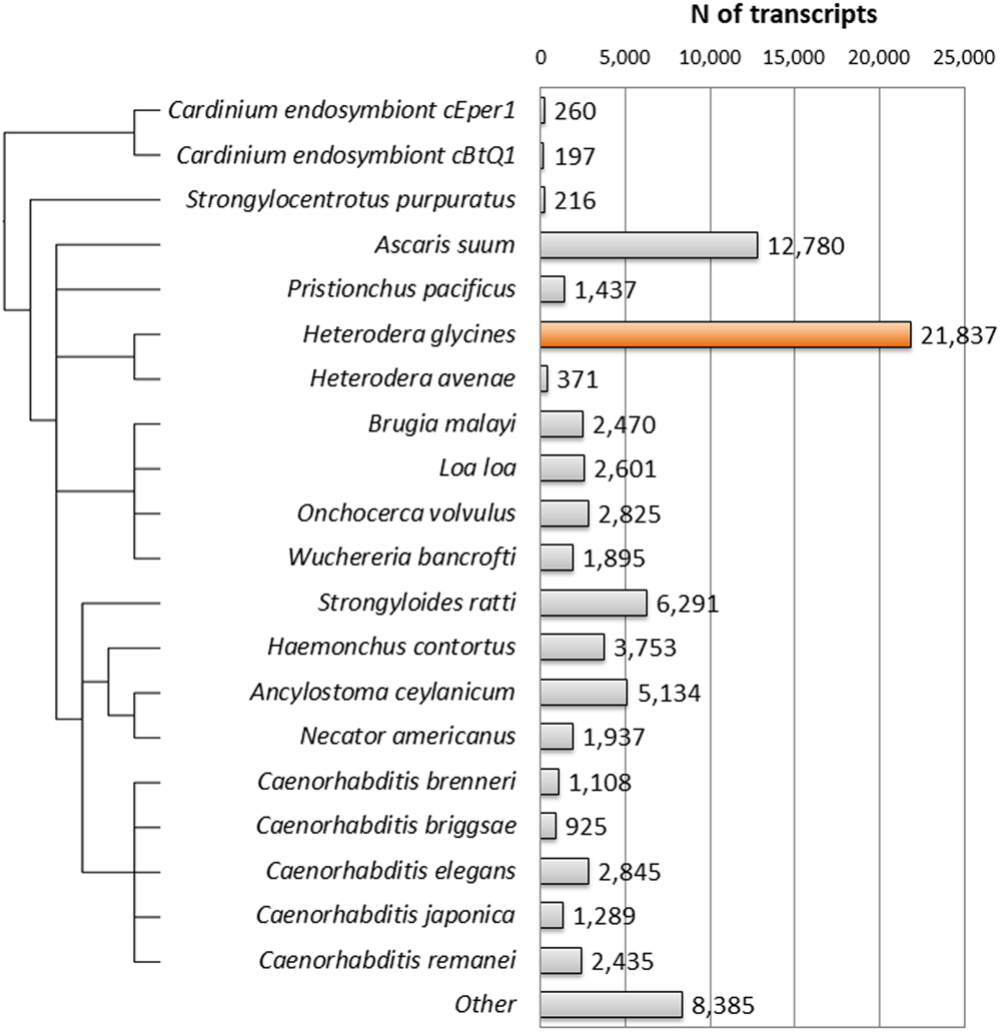
Species distribution of predicted homologues to *H. glycines*. Homologues were predicted using a BLASTX search against several protein databases at an e-value cutoff of 1e–5. The top 20 species with the most homologues are shown here. The resulting species evolutionary relationship was obtained from NCBI Taxonomy Browser^83^ and visualized using IcyTree^84^.

Transcripts from the *H. glycines* transcriptome were annotated following the Trinotate pipeline^25^. Transcripts were first compared to GenBank, Swissprot, and TrEMBL databases using BLASTX, resulting in a total of 66,601 (45.03%) out of the 147,910 transcripts annotated using an e-value cutoff of 1e–5 (S1 Table). When examining the species distribution of these significant hits, most transcripts hit to prior *H. glycines* database entries followed by animal-parasitic nematode species such as *Ascaris suum* and *Strongyloides ratti* (Fig. 1). In total, 1315 species are represented in the BLASTX results representing a broad variety of genera. Other species of note in the annotated transcripts include *Cardinium* endosymbionts of *Encarsia pergandiella* and *Bemesia tabaci* as well as several soybean cyst nematode associated viruses^26–31^. The virus sequences from the *H. glycines* PA3 population sequenced in this study are described by Ruark *et al*.^32^ and the endosymbiont-associated transcripts were characterized in more detail as described below.

**Figure 2:**
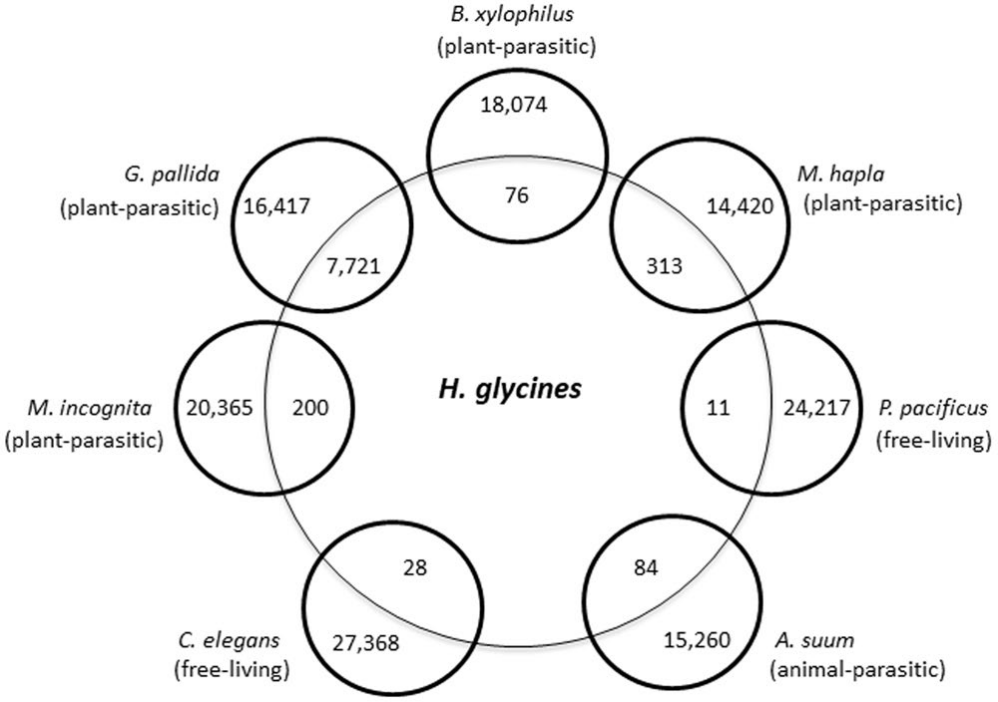
*H. glycines* orthologs in proteomes from sequenced nematodes with diverse feeding behaviors. The interior numbers represent predicted *H. glycines* proteins that only have orthologs identified in one of the seven other nematode species examined. Exterior numbers represent sequenced nematode proteins with no unique orthologs in the early parasitic *H. glycines* transcriptome.

Transcripts were further compared to several nematode species with sequenced and annotated genomes representing free-living, animal-parasitic, and plant-parasitic trophic groups to identify potential overlap and genes that are uniquely shared between SCN and one other nematode species. The *H. glycines* transcriptome uniquely shares 76 potential homologs with *Bursaphelenchus xylophilus*, 313 homologs with *Meloidogyne hapla*, 200 with *M. incognita*, and 7,721 with *Globodera pallida*. In addition, the transcriptome shares 11 homologs with the free-living nematode *Pristionchus pacificus*, 28 with the free-living nematode *Caenorhabditis elegans*, and 84 homologs with the animal-parasitic nematode *A. suum* (Fig. 2; S2 Table).

### Identification and GO annotation of endosymbiont-associated transcripts from the *H. glycines* transcriptome

**Figure 3:**
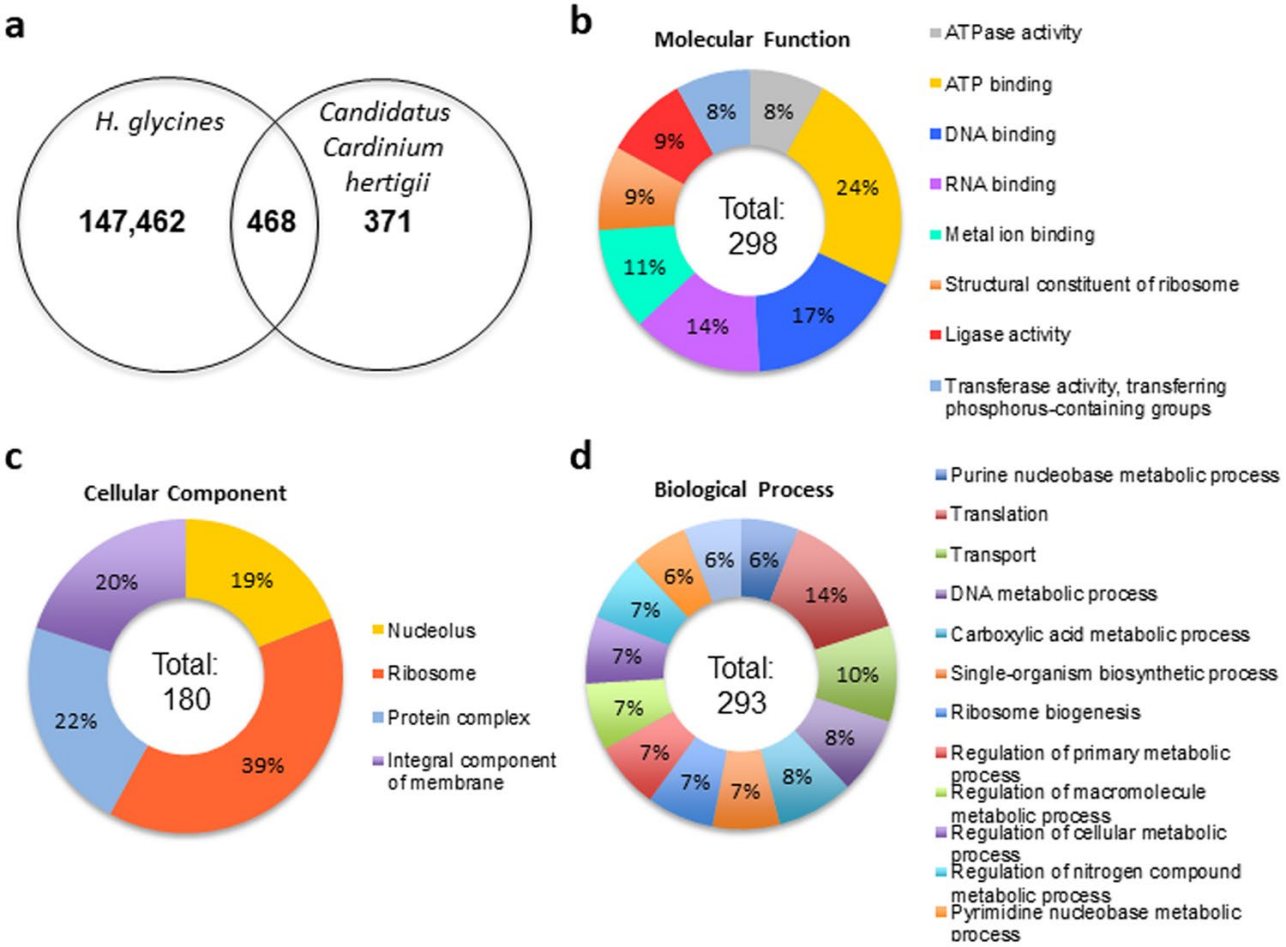
Identification and characterization of ‘*Candidatus* Cardinium hertigii’-associated transcripts within the *H. glycines* early life stage transcriptome. Transcripts from the *H. glycines* transcriptome were extracted and mapped against the proteome for *Candidatus* Cardinium hertigii to identify potential endosymbiont-associated transcripts, resulting in the identification of 468 of the 839 described proteins for this endosymbiont within the SCN early life stage transcriptome (**a**). Available gene ontology annotation was added to the endosymbiont-associated transcripts by BLAST2GO and grouped by the parent terms molecular function (**b**), cellular component (**c**), and biological process (**d**).

Prior microscopic analysis of SCN indicated the presence of a bacterial endosymbiont^28,31^. Within the early parasitic SCN transcriptome we identified 468 transcripts annotated as endosymbiont-associated transcripts, all of which were confirmed by BLASTX mapping to the *Cardinium hertigii* proteome (Fig. 3a; S3 Table). To further examine the potential functional significance of this inhabitant on SCN biology, GO terms were assigned to the 468 endosymbiont-associated transcripts using BLAST2GO, resulting in GO annotation of 328 of the 468 transcripts (S3 Table). Of those sequences with GO annotation within molecular function, the majority were involved in ATP binding, with 24% of the annotated transcripts falling into this category, followed by DNA (17%) and RNA (14%) binding (Fig. 3b). The cellular compartment represented by the greatest number of transcripts was the ribosome (39%) (Fig. 3c). The most significant biological processes represented among annotated transcripts were translation (14%) and transport (10%) (Fig. 3d).

### SCN stylet-secreted effector protein analysis

**Figure 4:**
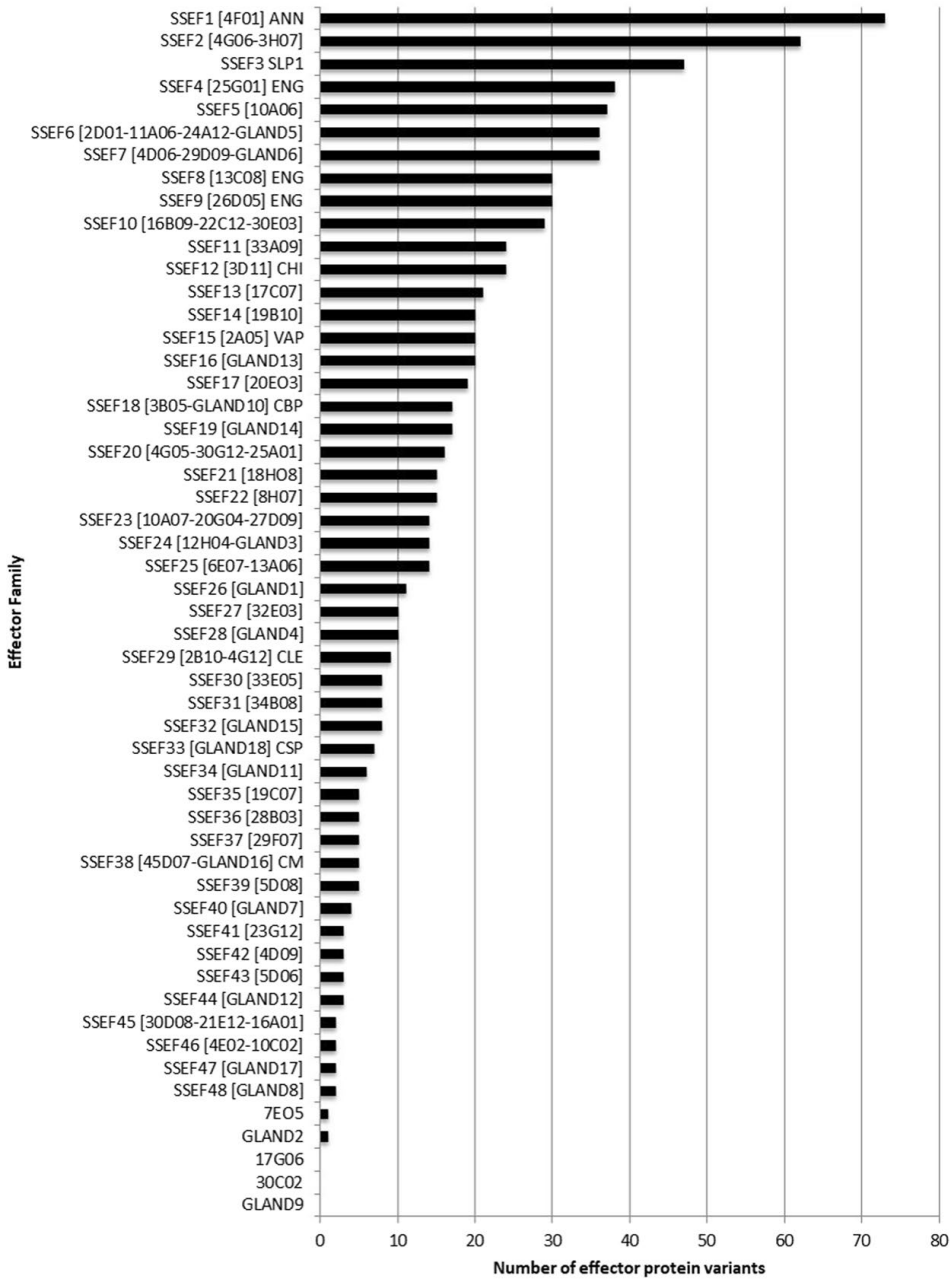
Variation of known effectors in the *H. glycines* early life stage transcriptome. Protein variants of previously published *H. glycines* effectors^17–19^ were identified using a BLASTP search at a 1e-5 cutoff and counted. Known effector sequences with >70% amino acid identity were grouped into stylet-secreted effector families (SSEF) to facilitate the analysis. Available functional annotation for effector families is indicated as follows: ANN = annexin-like; SLP1 = SNARE-like protein 1; ENG = endoglucanase; CHI = chitinase; VAP = venom allergen-like protein; CBP = cellulose-binding protein; CLE = CLAVATA3/EMBRYO SURROUNDING REGION (CLE)-like; CSP = circumsporozoite protein; CM = chorismate mutase.

Effector proteins originating in the esophageal gland cells and secreted through the stylet play critical roles in the SCN infection process. Therefore, we first examined the 72 previously identified stylet-secreted *H. glycines* effectors^17–19^ within the transcriptome. Of these, transcripts corresponding to each effector were identified using a BLASTN search, indicating that the transcriptome contained sufficient depth to detect expression of the known gland cell effector repertoire of SCN. An analysis of effector variation within the population was then performed. We first grouped the known effectors into stylet-secreted effector families (SSEFs) with greater than 70% sequence identity. To assess the level of variation of these known effectors within the sequenced *H. glycines* population, the predicted peptide sequences were mined for protein variants using BLASTP at a 1e–5 cutoff. Protein variants were identified for 69 of the 72 known effectors (Fig. 4). The remaining three (17G06, 30C02, and GLAND9) were found to have single nucleotide insertions and/or deletions leading to a frame shift in the predicted peptide, resulting in a completely different peptide compared to the reference sequence, and consequently were not examined for sequence variation. A wide scope of variation was identified, with some effectors having over 70 predicted protein variants across the population (e.g., annexin 4F01), while others were limited to a single, highly conserved protein sequence (e.g., 7E05, protein unknown function).

We then examined the expression of known SCN effectors during a compatible and an incompatible interaction to determine if the host environment influences the expression of any of these effectors. The effectors were split into two different groups (upregulated or downregulated) based on their expression pattern from the pre-parasitic second-stage juvenile (J2) stage to the parasitic J2 life stage and then compared across the two conditions (S4 Table). Most of the known SCN effectors followed the same pattern of expression across both comparisons, but the level of expression change was slightly reduced in the incompatible interaction. However, a subset of effectors exhibited an opposite trend of increased expression in the incompatible interaction, including members of SSEFs 1 [4F01], 9 [26D05], 17 [20E03], 22 [8H07], 45 [30D08, 21E12, 16A01], 39 [5D08], and 11 [33A09].

### Effector alternative splicing analysis

To analyze alternative splicing (AS) as a potential mechanism of effector variation, we used the 72 previously identified stylet-secreted *H. glycines* effector candidates^17–19^. Similar to the protein analysis of known SCN effectors, transcripts corresponding to each effector were identified using a BLASTN search in order to determine AS relationships. The major differences from the protein analysis were the use of a higher sequence similarity threshold (>85% identity) and the use of a gap penalty of 0. These two constraints were implemented to reduce false positives and improve true positives since gaps are expected to occur and should have a higher percent identity if AS occurs. In total, 395 AS transcripts were identified for the 72 previously known SCN effectors (Table 2), with the number of AS variants per each effector ranging from 1 to 38. Using these 395 AS transcripts, differential expression analysis was conducted to determine statistically significant AS transcripts for comparison between the ppJ2 and pJ2 life stages as well as between two different host interactions in the pJ2 life stage, an incompatible and compatible interaction. In total, 129 AS transcripts representing 44 known SCN effectors were determined to be statistically significant with respect to host interaction groups and 276 AS transcripts representing 58 known SCN effectors were statistically significant with respect to life stages, with 127 overlapping transcripts (98.4%) between stages (Table 2).

**Table 2:** Summary statistics for alternative splicing analysis of known SCN effectors.

	Known effectors	AS transcripts
Total	72	395
Significant for host interaction (compatible vs incompatible)	44	129
Significant for life stage (ppJ2 vs pJ2)	58	276

Alternative splicing analysis was performed on the previously published SCN effectors using the *de novo* transcriptome assembly. Splice variants were identified for known effectors and then analyzed for differential expression based on host interaction and nematode life stage.

To explore the effect that AS may have on protein function, functional domain analysis was conducted on the 395 AS transcripts. For this, we determined the changes in the functional domain architectures between specific AS isoforms. Since AS often alters the reading frame, all six reading frames were analyzed. Of the 72 effectors, 7 did not have any identified functional protein domains. In total, 513 protein functional domains for the remaining 65 effectors (7.9 domains per an isoform, on average) were identified using InterPro^33^. For the 395 AS transcripts, 910 protein functional domains were identified (2.3 domain, on average), with 108 transcripts with no functional domains identified. When considering each effector and their AS transcripts, 37 out of 65 effectors (57%) had AS events that altered the predicted domain architecture. The 395 transcripts included 198 architectures with no change, 247 with at least one added functional domain, and 78 with one or more functional domains deleted. We note that the numbers of domain architectures do not add up to 395 because in some cases a transcript belonging to one effector was identified as the AS transcript from a different effector.

**Figure 5:**
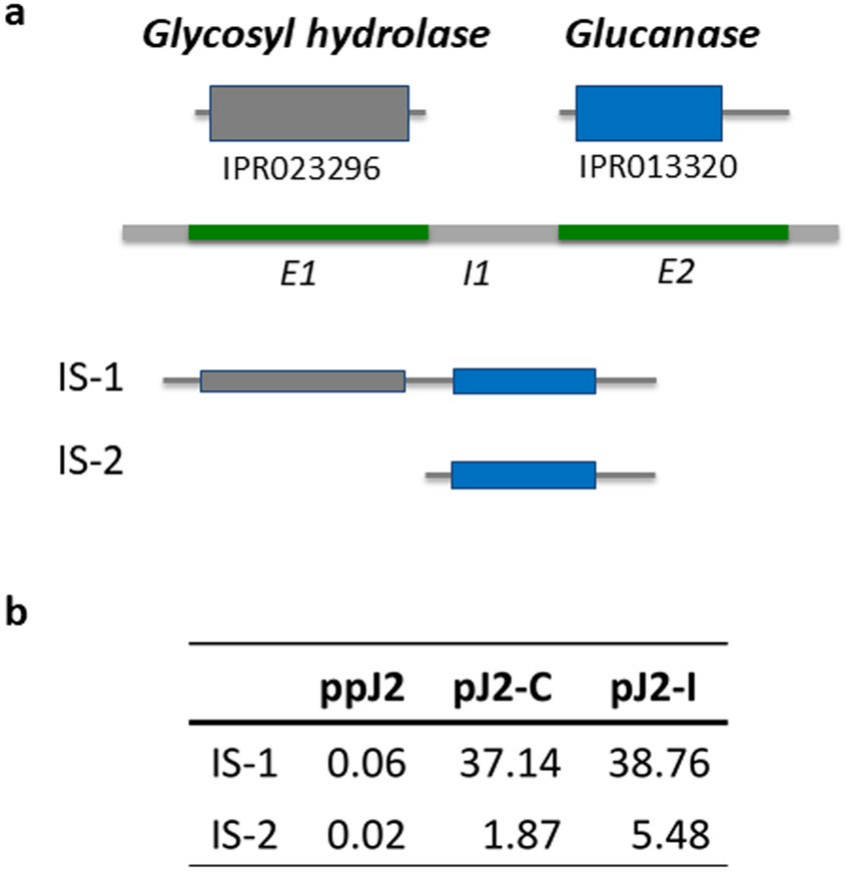
Gene structure, protein functional domain architecture, and isoform protein products for GLAND13. Domain architecture and the retained protein domains in each of two isoforms, IS-1 and IS-2 (**a**). Expression of each isoform (transcripts per million) in pre-parasitic second-stage juveniles (ppJ2) and parasitic J2 (pJ2) life stages during a compatible (**c**) or incompatible (I) host interaction (**b**). The first isoform was significant for life stage change (p-value is 9.07E-11), while the second isoform was significant for both life stage and host interaction changes (p-value 1.29E-5).

To analyze the functional changes in more detail, case studies of two effectors, GLAND13 and HgCLE (*Heterodera glycines* CLAVATA3/EMBRYO SURROUNDING REGION-like), were considered together with their AS transcripts. GLAND13 was chosen to demonstrate a simple example of a clear association between a protein function and AS variation due to the differentially spliced isoforms. On the other hand, HgCLE was chosen to demonstrate the structural and functional complexity that could be invoked through alternatively spliced isoforms. The architecture of the GLAND13 protein was predicted to have two functional domains that corresponded almost exactly to the two exons (Fig. 5a). These two protein domains were associated with glycosyl hydrolase, a five-blade beta propeller domain, and concanavalin A-like lectin/glucanase protein domain (InterPro IDs: IPR023296 and IPR013320, respectively). Both of the functional domains are known to associate with metabolism. Our *de novo* AS analysis determined two different transcripts associated with GLAND13. The primary transcript included both protein domains, while the secondary transcript had exon 1 spliced out. It is possible for the reading frame to be altered if an AS event modifies the beginning of the gene. However, in our case the reading frame was preserved, which caused a removal of the glycosyl hydrolase domain, while leaving intact the glucanase domain. The functional implications of this removal are yet to be experimentally characterized. However, it was clear from the analysis that the primary transcript was important for life stage and was upregulated in the parasitic stage (p-value is 9.07E-11). Additionally, the secondary transcript was important for both life stage and host interaction (p-value 1.29E-5, Fig. 5b). This transcript was upregulated in the parasitic stage, but to a greater extent in nematodes infecting a resistant host plant.

**Figure 6:**
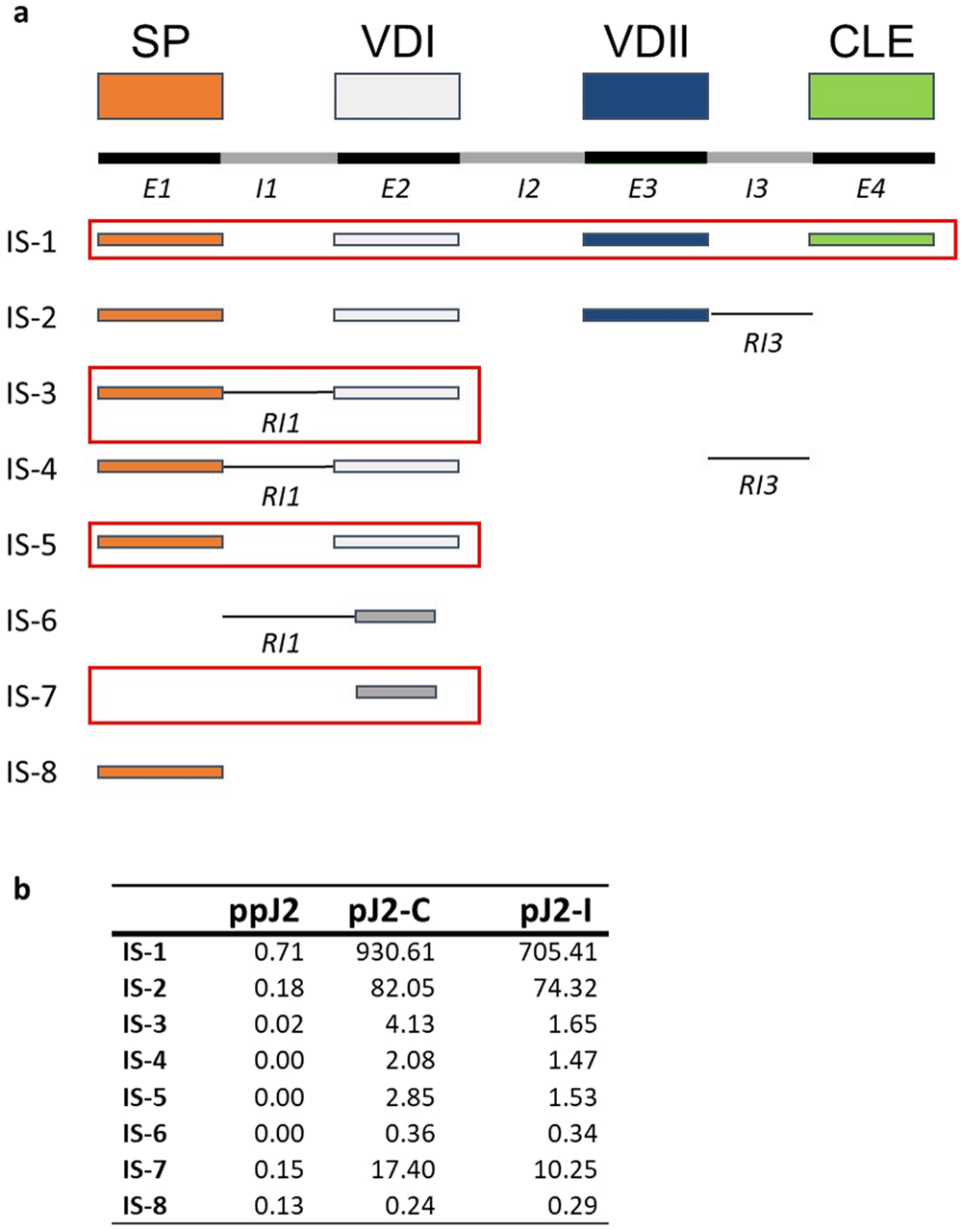
Gene structure, protein functional domain architecture, and isoform protein products for HgCLE2. Domain architecture and the retained protein domains in each of eight isoforms, IS-1 and IS-8 (**a**). Shown in red are the retained introns. Each retained intron was modified as a result of AS. Dark grey boxes correspond to a modified VD1 domain due to AS. Expression of each isoform (transcripts per million) in pre-parasitic second-stage juveniles (ppJ2) and parasitic J2 (pJ2) life stages during a compatible (**c**) or incompatible (I) host interaction (**b**). Red boxes highlight transcripts that were statistically different for both life stage and host interaction groups.

HgCLE genomic architecture includes four exons that were consistent with four functional subunits: signal peptide, variable domain I, variable domain II, and the CLE domain^34^. The N-terminal signal peptide is important for secretion of the peptide out of the nematode esophageal gland cell while variable domain I has been shown to function in targeting of the effector within the host plant cell^35^. The HgCLE effector family [2B10-4G12] contains two known members with high levels of sequence conservation at the amino acid level, the only differences existing within the variable domains. The CLE domain is processed to release a small peptide that functions within the host plant as a ligand mimic^34^. Based on the AS analysis, there were 8 transcripts associated with HgCLE. To improve the AS analysis, the corresponding HgCLE2 genomic DNA sequence was retrieved from NCBI GenBank (GenBank ID: FJ503005.1) and compared with these 8 transcripts (Fig. 6a). While the genomic sequence was obtained from a nematode population that was different from the one used in this study, it was expected that there would be a significant sequence similarity between the gene sequence and the AS isoforms if there were AS events associated with intron retention. Transcript 1 corresponded to the full sequence of HgCLE2 retaining all four exons. Transcript 2 included exon 1–3, but retained intron 3 and lacked exon 4, which was associated with the CLE domain. Transcript 3 contained exon 1 and 2, but retained a modified version of intron 1. Transcript 4 was similar to transcript 3 except intron 3 was retained. Transcript 5 included just exon 1 and 2. Transcript 6 included modified versions of intron 1 and exon 2. Transcript 7 included a modified version of exon 2. Transcript 8 included exon 1. With respect to the differential expression analysis, transcript 1, 3, 5 and 7 were statistically significant (p-values ranging from 5.50E-04 to 9.638E-05) for life stage and host interaction, transcripts 2 and 4 were statistically significant (p-value 5.27E-07 and 4.96E-10) only in regards to the life stage, and transcripts 6 and 8 were not differentially expressed between any group (Fig. 6b).

### Novel effector prediction

**Figure 7:**
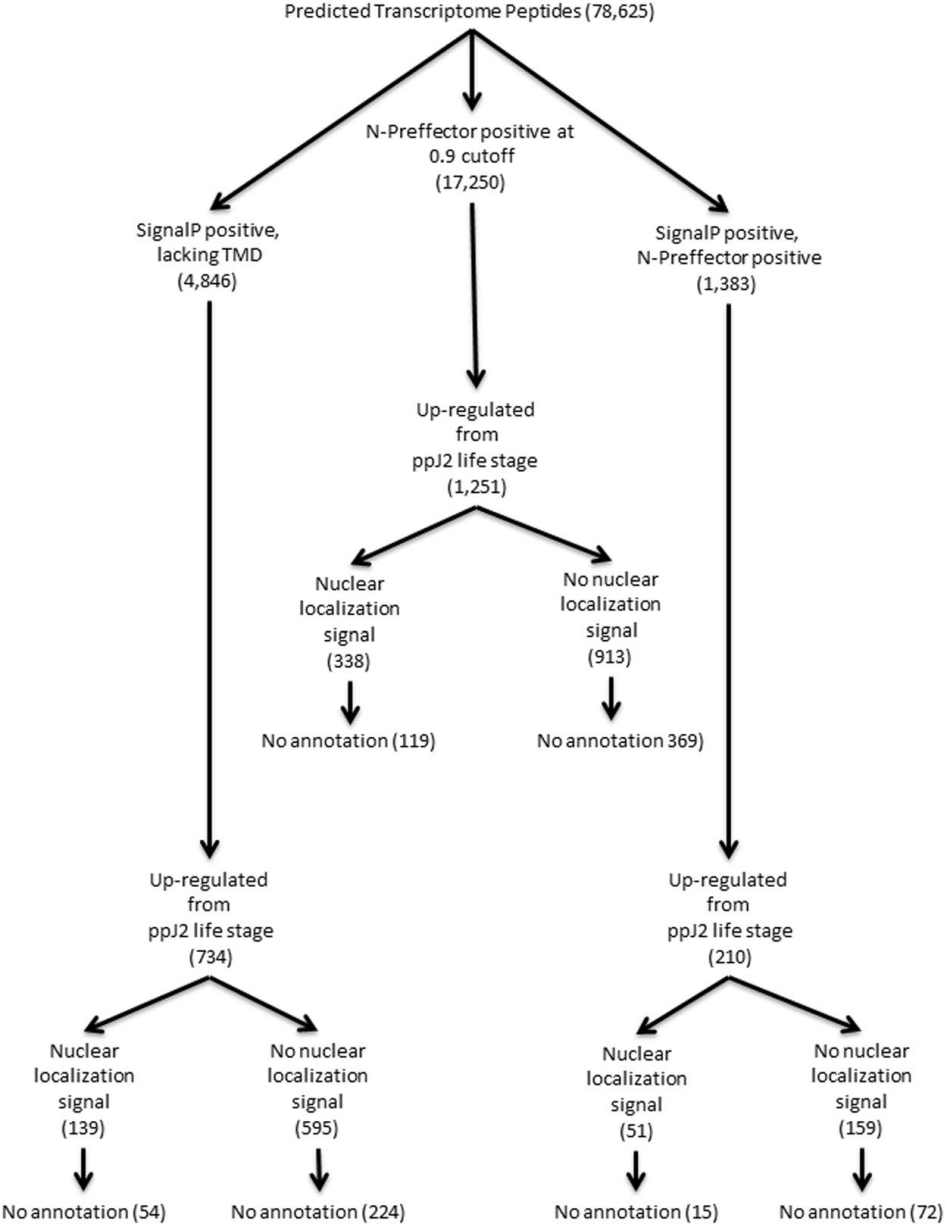
Secreted effector protein prediction in the early life stage transcriptome of *H. glycines*. Predicted peptides from the transcriptome were put through two separate pipelines to identify candidate effectors. One pipeline utilized prediction of a signal peptide and lack of a predicted transmembrane domain (TMD) while the other utilized N-Preffector, a machine learning algorithm. Numbers shown here are predicted peptides remaining after each step in the pipeline.

We then performed a comprehensive effector analysis on the SCN transcriptome. Effectors were predicted using two separate pipelines, and then the results were compared to determine the overlap of each pipeline (Fig. 7; S5 Table). The first of these pipelines relies on the presence of a signal peptide and follows the method used in previous studies for the prediction of putative stylet-secreted effectors^18,19^. This pipeline predicted 4,846 putative effectors. To identify putative new effectors with higher confidence, we focused on genes upregulated from the pre-parasitic J2 to parasitic J2 life stage and analyzed the sequences for the presence of a nuclear localization signal (NLS). A NLS combined with an N-terminal signal peptide is a strong indicator for localization of these effectors into host cell nuclei where they can play a variety of functions including regulation of plant defense responses^20,36^. Following these filtering steps, this pipeline predicted 734 effector candidates, including 139 nuclear localization signal (NLS)-positive effector candidates up-regulated from the pre-parasitic J2 to the parasitic J2 life stage (Fig. 7; S5 Table). The 72 known SCN effector proteins, known to contain signal peptides, were re-discovered at a rate of 74% using this pipeline. This pipeline is reliant upon the presence of a N-terminal signal peptide, which may not be present if the N-terminus is absent from the transcript. This is reflected in the fact that several known SCN effectors were not recovered despite their nucleotide sequences being present within the transcriptome. A second pipeline independent of the presence of a signal peptide was performed using N-Preffector, a machine learning algorithm trained on known nematode and bacterial effectors. The N-Preffector-based pipeline predicted 1,251 putative effectors, including 338 NLS positive effector candidates up-regulated from the pre-parasitic J2 to the parasitic J2 life stage (Fig. 7; S5 Table). In this pipeline, 67% of the known SCN effectors were re-discovered. When the two pipelines were compared, 210 effector candidates were found to be overlapping, including 51 NLS positive candidates (Fig. 7; S5 Table). Many of these sequences have little or no annotation available. Among those sequences with available annotation are many homologs of effectors from other plant-parasitic nematodes that were not previously identified or characterized in *H. glycines* (S6 Table). These include effectors such as glutathione synthetase^37^ and members of the SPRYSEC family^38^.

## Discussion

In this study, we sequenced the transcriptome of the early life stages of the plant-parasitic nematode *Heterodera glycines*, including the infective (pre-parasitic) second-stage juvenile (J2) life stage and the parasitic J2 life stage in two different host conditions, resistant and susceptible. We then carried out a *de novo* transcriptome assembly with an emphasis on assessing the level of variation of known effectors within a single population and identifying novel secreted effectors within *H. glycines* that may play important roles in establishing a parasitic interaction with its host, soybean. The resulting transcriptome from these samples consisted of nearly 150,000 transcripts encoding 78,625 predicted proteins. There are several possible explanations for the large number of transcripts identified. First, to generate the transcriptome a large population of nematodes was sequenced. The inherent genotypic heterogeneity present within the population may lead to many variants of the same gene being represented within the transcriptome. In addition, following transcriptome assembly no expression threshold was applied. This was done to capture any rare or lowly expressed transcripts within the population. Of the 147,910 transcripts contained within the *H. glycines* transcriptome, 66,601 (48%) were annotated based on BLAST homology. Many of these potential homologues existed in other nematode species, including plant- and animal-parasitic nematodes. In addition, some transcripts showed homology to a bacterial endosymbiont from the genus *Cardinium*, of *Encarsia pergandiella*, a parasitic wasp, and *Bemesia tabaci*, a whitefly. Previous work identified this endosymbiont and characterized it as *Candidatus* Paenicardinium endonii, later renamed to *Candidatus* Cardinium hertigii^28,39^. However, little is known about the function of this endosymbiont and what role it may play, if any, in plant parasitism. Related endosymbionts found in insects and arachnids have been shown to have prominent impacts on their hosts, leading to changes in host reproductive capacity and also modulating host immunity^40^. To better understand the function of a putative endosymbiont in *H. glycines* all transcripts associated with this endosymbiont were identified and extracted from the transcriptome, representing a majority of the characterized sequences for this endosymbiont. Those transcripts identified were primarily associated with metabolic processes, which may contribute to both nematode and endosymbiont metabolism. Further studies into the function of this endosymbiont and any effect on parasitism removing the endosymbiont has will be vital in elucidating what role it plays inside the nematode. In addition to a bacterial endosymbiont, several putative homologs from viruses were also identified. Previously, researchers found representative viruses from the *Bornaviridae, Rhabdoviridae*, and *Bunyaviridae* families contained within *H. glycines*^26,32^. Thus, there appears to be a significant microbial community active within *H. glycines* that has until now remained largely unexplored. Further examination of these organisms could reveal vital connections that can be exploited for improving resistance against SCN.

Stylet-secreted effectors represent a key component of the plant-nematode interaction, serving a wide variety of functions required for successful invasion and establishment of the nematode feeding site. Previous studies have identified a suite of these effectors using microaspiration techniques to isolate the contents of the esophageal gland cells where these genes are expressed^18,19^. These studies then prioritized potential effectors based on those sequences possessing a signal peptide and lacking a transmembrane domain. Despite previous knowledge about the effector repertoire of SCN, very little is known about the structure of these sequences within a population, specifically how these sequences vary from one individual to another. To address this question, we undertook an effector variation analysis within the transcriptome, identifying putative variants of known effectors and examining their level of variation within the population. Within the SCN transcriptome, predicted sequence variants of known effectors ranged from over 70 (effector 4F01) down to one (effectors 7E05 and GLAND2). This effector variation may be a result of different alleles being present in the population and/or reflect variation in the copy numbers of genes encoding related effectors. The level of variation of these effectors is likely related to the function of the effector in question. For example, a highly variable effector such as 4F01 may be under constant selection pressure to avoid host recognition, resulting in a wide level of variation across the gene pool. A prior study demonstrated that 4F01 might function as a mimic of host plant annexins to promote successful plant infection^41^. By contrast, effectors with limited variation across the population are likely constrained by their function. It would be interesting to see how a highly virulent population or a field population compares to the highly inbred population used here for sequencing. Certain effectors may be expanded or reduced depending on the population and host selection pressure. Effectors with a very low number of variants across populations may represent key elements of infection that could be targeted for further study in the attempt at identifying a novel source of broad spectrum SCN resistance.

One potential mechanism of gene regulation that can introduce variation into genetic sequences is alternative splicing. Previous work has identified alternative splicing in stylet-secreted effectors from SCN on an individual basis and demonstrated that expression of these variants was impacted by the life stage of the nematode^42,43^. As sequence data become available for plant-parasitic nematodes, these types of analysis can be expanded to larger scales. For example, a comprehensive analysis of alternative splicing events conducted across the effector complement of the potato cyst nematode *G. pallida* using the sequenced genome found that 38% of these genes undergo alternative splicing and that certain families of effectors show increased occurrence of splicing relative to others^44^. With the early parasitic transcriptome generated in this study we were able to perform a large-scale alternative splicing analysis on the known effectors of SCN and identified significant changes in the expression of alternatively spliced transcripts for a majority of the effectors between the ppJ2 and pJ2 life stages as well as between compatible and incompatible host interactions. Changes in effector splicing across life stages as the nematode begins infection may be important for altering the protein function or activity to facilitate migration and establishment of the nematode feeding site. We then examined alternative splicing of effectors between a compatible and an incompatible host interaction, identifying a smaller subset of effectors with significant expression changes between these two conditions. These splice variants may be useful once again for altering function and activity of the effectors, potentially after being triggered by perception of host resistance by the nematode. By expressing an alternate version of the effector sequence, the nematode may avoid direct recognition of the host or recognition of the function that effector performs. Once additional populations of SCN have been sequenced it will be interesting to see whether these splice variants are involved in virulence on other sources of SCN resistance and if these can be targeted to improve overall resistance to this pathogen.

We also mined the early parasitic transcriptome to identify additional effectors expressed within *H. glycines* using the SignalP predictive tool, as well as a novel pipeline called N-Preffector. The use of N-Preffector allowed for the identification of an entirely new class of effectors not necessarily containing a signal peptide. Examples of secreted effectors lacking a signal peptide have been shown in other plant-parasitic nematode species such as *G. rostochiensis*, where they have been shown to play a role in disrupting host reactive oxygen species production^45,46^. These effectors may contain a previously unknown secretion signal or utilize a novel secretion pathway in order to be secreted. Between the two pathways utilized for effector discovery, 86% of known SCN effectors were re-discovered within the early parasitic SCN transcriptome. The remaining 14% were not re-discovered either due to truncated sequences relative to the reference sequence or a change in the predicted protein sequence between the transcriptome and reference sequence. It is interesting to note that 47% of these effectors were identified by both pipelines, but included different effectors. This illustrates the potential advantage of using both pipelines to accurately detect all possible effectors including those that one pipeline may not identify. The signal peptide-dependent method is excellent at predicting putative effectors, but misses out on transcripts that may be truncated or simply lack the signal peptide, which can be complemented using the N-Preffector pipeline. It should also be noted that in this study an expression change between life-stages was used as a parameter for effector prediction and to limit the overall number of false positives. For this reason, the possibility exists that some putative effectors with very low expression levels may have eluded discovery. One example is HgCLEB, which is expressed at low levels and therefore was not discovered in the effector pipeline, but later identified using a targeted search of the transcriptome^47^.

The novel effector candidates identified by these two pipelines represent a set of genes for downstream expression and functional analysis to investigate the interaction between SCN and soybean. Many of these sequences have little or no annotation available, much like the original gland isolated effector sequences obtained for *H. glycines*^17–19^. These novel effector sequences may play pivotal roles in nematode parasitism and will require more in depth functional studies to determine their function. Among those sequences with available annotation are many homologs of effectors from other plant-parasitic nematodes that were not identified or characterized in *H. glycines* previously. Included in this category are genes such as the glutathione synthetase family, the novel *G. rostochiensis* effector E9, and candidates showing homology to the SPRYSEC family of effectors from *G. rostochiensis*. Glutathione synthetases have many potential roles in the interaction between the nematode and host plant. In the interaction of the root-knot nematode *M. incognita* it was found that glutathione is needed for successful infection of the host plant *Medicago truncatula*^37^. In addition, glutathione synthetase genes were found to be greatly expanded in the genome of the potato cyst nematode *G. pallida*, where these genes are theorized to be involved in protection of the nematode from antioxidant proteins as well as potentially in nematode nutrition^8^. Several transcripts annotated as glutathione synthetase also contained a secretion signal, something that differentiates them from glutathione synthetases found in animal parasites that may function within the nematode. The putative effector E9 has been identified in both *G. rostochiensis* and *G. pallida* and was confirmed to be expressed in gland cells via *in situ* hybridization^44,48^. Thus far little is known about the function of the E9 effector, other than it being expressed in the gland cells of *Globodera* species. The SPRYSEC effectors on the other hand have been heavily investigated in the *Globodera*-tomato pathosystem, with demonstrated roles in the suppression of plant immune responses^38,48^. To date, SPRYSEC effectors have not been identified in the genome sequence of root-knot nematodes^8^ however, entries in non-redundant sequence databases suggest they may be present in other cyst nematodes and lesion nematodes^49^. Thus, these could be very interesting candidates for comparative analysis across virulent populations of SCN to determine whether or not they play the same role as in *Globodera spp*. Another effector candidate of note is a putative secreted calreticulin. A calreticulin secreted by *M. incognita* is necessary for successful infection and may play a role in suppression of plant defenses; functions that may be retained in *H. glycines*^50^. Another nematode effector homolog group identified in the transcriptome involved in suppression of host defenses are the C-type lectins (CTLs) from *Rotylenchus reniformus*. These effectors were identified in the *R. reniformus* transcriptome and subsequently shown to be expressed in the hypodermis of parasitic stages of the nematode^51^. It is hypothesized that these effectors are involved in protecting the nematode from environmental stress. While these homologs are all predicted to have the same function in *H. glycines* as their originating species, further functional characterization is necessary to confirm this.

Interestingly, we identified several effector candidates with sequence similarity to proteins originating in plants and other organisms. These included multiple effector candidates with homology to members of the plant RING/U-box superfamily of proteins. These proteins are typically involved in protein modification and regulation of plant pathways, including defense responses and regulation of cell death^52^. Nematode mimics of these proteins may be involved in manipulation or suppression of host defense pathways in order to allow successful establishment of the feeding site. Among the identified effector candidates are also several homologs related to plant metabolism and cell wall degradation. These included arabinosidase, fructosidase, glycoside hydrolase, and expansin. These cell wall modifying proteins have been shown to aid in the loosening and degradation of polysaccharides present in the plant cell wall^53–55^ and have been identified from other plant-parasitic nematodes where they play a crucial function in migration and establishment of the nematode feeding site^56,57^. Therefore, these plant mimics all represent avenues of study to be pursued in order to better understand the interplay between SCN and its plant host, soybean.

In conclusion, a *de novo* transcriptome of the pre-parasitic and parasitic second-stage juvenile life stages of *H. glycines* has been generated, annotated, and comprehensively mined for putative effector sequences. Within this transcriptome novel effector candidates were identified utilizing a new prediction tool not reliant on sequences possessing a signal peptide, N-Preffector. In addition, the level of variation of previously identified *H. glycines* effectors was examined for the first time at the population level and identified highly conserved and highly variable effectors. Finally, this transcriptome provides a useful genetic resource that will aid in annotation of the SCN genome. Combining these data will provide insights into the biology of SCN with the hopes of discovering new ways to combat this pathogen.

## Materials and Methods

### Nematode cultivation and isolation

The SCN inbred population PA3 (HG Type 0) was propagated under greenhouse conditions on susceptible soybean Williams 82 or EXF63. Freshly hatched pre-parasitic second-stage juveniles (ppJ2) were inoculated onto 10-day old seedlings of the susceptible host or the resistant host (cv. Forrest) and the inoculated plants were placed in the greenhouse. The remaining ppJ2 nematodes were pelleted by centrifugation and flash-frozen in liquid nitrogen and stored at −80 °C prior to RNA isolation. Five days post-inoculation, parasitic second-stage juveniles (pJ2) nematodes were isolated from the roots by blending the roots for 30 s in a kitchen blender. Following this, the root homogenate was poured over a nested stack of sieves with pore sizes of 850 µm, 250 µm, and 25 µm before purifying the nematodes from the sample using sucrose centrifugal flotation^58^. Samples were frozen in liquid nitrogen and stored at −80 °C prior to RNA isolation.

### RNA isolation and sequencing

RNA was isolated from frozen nematode pellets using the PerfectPure Fibrous Tissue Kit (5Prime) and a modified version of the manufacturer’s extraction protocol. Tissue was homogenized in 30 second intervals in the provided lysis solution containing 0.5 µM TCEP using a bead beater and 1.0 mm zirconia beads, followed by a 30 second incubation on ice. This was repeated three times. The sample was centrifuged briefly at room temperature before transferring the supernatant to a fresh tube. Following lysis and homogenization, 10 µl of the provided Proteinase K was added and the sample was allowed to incubate on ice for 10 minutes, after which the manufacturer’s protocol for RNA purification was followed. RNA quality was determined using a Fragment Analyzer (Advanced Analytical) and quantified using a Qubit Fluorometer prior to library preparation. RNA-seq libraries (ppJ2, pJ2 infecting susceptible host, pJ2 infecting resistant host) were constructed using the TruSeq mRNA Stranded Library Prep Kit (Illumina) and sequenced on the Illumina HiSeq. 2500 platform in a paired-end manner (2 × 100 for ppJ2 and pJ2-Compatible samples and 2 × 50 for pJ2-Incompatible sample). Library preparation and high-throughput sequencing services were performed at the University of Missouri DNA Core Facility. Three biological replicates of each sample were sequenced.

### *De novo* transcriptome assembly

Prior to assembly, raw reads from these libraries were filtered using Trimmomatic^59^ to remove low quality reads. The remaining reads were paired and orphan reads discarded. High quality paired-end reads were used as input for transcriptome assembly. *De novo* transcriptome assembly was completed using the de Bruijn graph-based tool Trinity^60^. As part of the assembly process, an *in silico* read normalization step was performed. Assembly quality was then assessed by mapping raw reads back to transcripts using Bowtie2^61^ at default parameters.

### Transcriptome annotation and quantification

The transcriptome was annotated following the established Trinotate pipeline^60^. Homology searches were performed against the protein sequences contained in Genbank^62^ and UniProt^63^ databases using BLASTX at an evalue cutoff of 1e-5^64^. Transcripts were translated into protein using TransDecoder, a component of Trinity^60^. HMMER and Pfam databases were used to predict protein domains contained within each transcript^65,66^. Presence of a signal peptide was determined using SignalP version 4.0 and TMHMM version 2.0 was utilized to identify predicted transmembrane domains^67,68^. The resulting annotation information was then combined and pooled into a SQLite database. In addition, sequenced nematode genomes were leveraged to identify potential homologs within the transcriptome. For this, predicted protein datasets from the genomes of *Bursaphalenchus xylophilus, Meloidogyne hapla, Meloidogyne incognita, Globodera pallida, Pristionchus pacificus, Ascaris suum*, and *Caenorhabditis elegans* were downloaded from WormBase (http://ws204.wormbase.org/) and used^69^. BLASTP hits from the *H. glycines* transcriptome with e-values less than 1e–5 were considered potential homologs. Lists of potential homologs from each of the seven species examined were then compared and contrasted to determine uniquely shared homologs between the sequenced nematode and *H. glycines*.

For quantification and differential expression analysis, reads from the libraries used for assembly were mapped and quantified using RSEM^70^ to determine transcript abundance. RSEM was utilized as it has been shown to correlate well with RT-qPCR measurements and produce expression values with high accuracy^71^. Following quantification, differential expression analysis was conducted using edgeR^72^, identifying all genes with a minimum 4-fold expression difference and under a p-value cutoff of 0.001 between any of the samples.

### Identification of endosymbiont sequences within the *H. glycines* transcriptome

The entire *de novo* early parasitic transcriptome for *H. glycines* was mined for transcripts related to the endosymbiont “*Candidatus* Cardinium hertigii”. All transcripts annotated with the species designation ‘Cardinium endosymbiont’ were extracted from the transcriptome and combined into a file. A database was then constructed from the complete proteome of the closest available sequenced bacterial isolate, *Cardinium hertigii* cEper1 isolated from *Encarsia pergandiella*^29^. Then all putative *Cardinium*-associated sequences were mapped against the proteome database using BLASTX at an e-value cutoff of 1e–5 to confirm their identity as putative endosymbiont-associated transcripts. The resulting transcripts were then used for gene ontology analysis.

### Gene ontology analysis of endosymbiont-associated sequences from the *H. glycines* transcriptome

Gene ontology (GO) analysis was performed to identify the putative function of endosymbiont-associated sequences within SCN. To do this endosymbiont-associated sequences from the SCN transcriptome were used in the research tool BLAST2GO^73^. This tool uses a similarity searches to assign GO annotation to sequence data lacking well-characterized GO annotation. In BLAST2GO BLASTX was performed at an e-value cutoff of 1e-5 and the top available BLAST hit used to pull available GO annotation. Once available GO annotation was assigned to the 468 endosymbiont-associated transcripts the results were examined for their potential role in SCN biology.

### Variation of known SCN effectors

The protein sequences for the 72 known SCN effector sequences^17–19^ were aligned using MUSCLE^74^ and then a maximum likelihood tree was constructed based on sequence homology in MEGA7^75^. MUSCLE (multiple sequence comparison by log-expectation) is a high accuracy tool for protein alignment. Effectors with bootstrap values greater than 50 were grouped into stylet-secreted effector families (SSEFs). Predicted transcript peptide sequences from the SCN transcriptome were then mapped to known SCN effector protein sequences using BLASTP at an e-value cutoff of 1e–5 and quantified for each known effector. Variants of known SCN effectors in a SSEF were pooled for quantification.

### Effector alternative splicing analysis

*De novo* alternative splicing analysis represents a challenging task since a complete *H. glycines* genome is not available to assess exon and intron relationship^73^. However, it is possible to associate known genes of interest and build associated relationships to infer alternative splicing events by comparing known regions of overlap and extract exons associated with specific alternative splicing isoforms. This alternative splicing analysis relies on the transcripts that are assembled with the Trinity pipeline^25^. The alternative splicing quantification is then carried out with the *kallisto* tool^76^ using the preprocessed reads and the pseudo alignment on the assembled transcripts, which allows the analysis to be computationally more efficient, without losing its quality. Using these quantified transcripts, *sleuth* tool was employed to determine statistically significant differentially expressed transcripts^77^. From the list of 72 known SCN effector genes, the inferred alternative splicing relationship is built based on significant overlap between effector sequences and transcripts, defined by sequence identity of greater than 85%. The overlap and sequence identity are determined using the BLASTN tool, with the gap penalty parameter set to 0^78^. This high sequence identity threshold is used because a true alternative splicing event is expected to have a significant exon overlap between the effector sequences and transcripts. The reason that a higher identity threshold is not used is because the SCN population used as a source for the effector genes is different from the SCN population used as a source for transcriptomic data obtained in this study. Combining the high sequence identity threshold and zero gap penalty in the BLASTN search, thus, allows for alternative splicing events of known exons from the effector genes to be identified, while not allowing the discovery of new relationships. New relationships that will be missed due to the data and methodology limitation are primarily the intron retention events and require the assembled genome as a reference. Using the identified associated alternative spliced transcripts, protein functional analysis is done by predicting the domain architectures and characterizing protein domains using InterPro^79^. Since it is expected for the reading frame to change, all 6 reading frames (forward and reverse) are assessed for the domain architectures and protein functions. In summary, this approach allows one to functionally characterize the differential expression changes for alternative spliced transcripts. These functionally characterized differentially expressed transcripts were compared between different nematode life stages and host interactions.

### Effector prediction

The effector prediction pipeline started with all predicted peptides from the SCN transcriptome. First, sequences represented in the gland cell transcriptome were subjected to two different prediction tools: SignalP^67^ and N-Preffector, developed in this study. For the SignalP-based prediction, peptides were run through SignalP 4.0 and TMHMM^68^ to predict signal peptides and transmembrane helices, respectively. Predicted peptides containing a signal peptide and lacking a transmembrane domain were then filtered based on their expression between the ppJ2 and pJ2 life stages of the nematode, with those peptides showing a minimum 4-fold up-regulation into the pJ2 life stage retained. Finally, nuclear localization signals were predicted using NLStradamus^80^. For N-Preffector based prediction, predicted peptides were run through a machine-learning algorithm trained on 72 known *H. glycines* effector sequences and 150 known non-effector sequences from *H. glycines* in addition to the original sequences (gram negative bacteria) in which the Preffector model was trained^81^. For each protein sequence, N-Preffector calculates a vector of length-invariant features; the feature vector is then used as an input for the classification model. Feature categories that were considered are: residue composition, sequence/structure information, and physico-chemical properties of proteins. To select highly correlated features with the class and not correlated with each other, Preffector utilizes the correlation-based feature selection (CFS) method^82^. Our goal was to minimize the number of proteins erroneously misclassified as effectors, *i.e*., false positives, while trying to maximize the number of predicted real effectors, using the same exact protocol utilized in Preffector. N-Preffector achieves this through a more stringent classification criterion. Given an SVM model *M* and a training data of size *n*, for each training example *x*_*k*_, let ${f}_{k}\in [-1,-1]$ be its decision value predicted by the SVM model, and ${y}_{k}\in \{+1,-1\}$ be its true annotation of being an effector or non-effector. Given the SVM model *M*, the prediction probability for a training example *x*_*k*_ is defined as

1 $${p}_{k}^{(i)}=\frac{1}{(1+\exp ({A}^{(i)}{f}_{k}^{(i)}+{B}^{(i)}))}.$$

The coefficients *A*^*(i)*^ and *B*^*(i)*^ are estimated during the SVM training process by minimizing the log-likelihood function. Those peptides predicted by N-Preffector at or above a 0.9 confidence score cutoff were then filtered based on expression, retaining peptides with a minimum 4-fold up-regulation from the ppJ2 to the pJ2 life stages. Nuclear localization signals were then predicted using NLStradamus for the remaining peptides^80^.

### Data Availability

Raw sequence reads are available under the Short Read Archive (SRA) accession no. SRP122521. This Transcriptome Shotgun Assembly project has been deposited at DDBJ/EMBL/GenBank under the accession GFZZ00000000. The version described in this paper is the first version, GFZZ01000000.

## Electronic supplementary material

Supplementary Information

Table S1

Table S2

Table S3

Table S4

Table S5

Table S6
