# Supplementary material for: Novel global effector mining from the transcriptome of early life stages of the soybean cyst nematode Heterodera glycines
Source: Sci Rep. 2018 Feb 6;8:2505. doi: 10.1038/s41598-018-20536-5 (PMC5802810; doi:10.1038/s41598-018-20536-5)
Supplement: Supplementary file 1 — Supplementary Information [file 41598_2018_20536_MOESM1_ESM.pdf]

Supplementary Information

Novel global effector mining from the transcriptome of early life stages of the soybean cyst nematode *Heterodera glycines*

Michael Gardner, Andi Dhroso, Nathan Johnson, Eric L. Davis, Thomas J. Baum, Dmitry Korkin, and Melissa G. Mitchum

Supplementary Table 1. Annotation summary for the transcripts. Annotation generated through the Trinotate pipeline was condensed into a single database with information provided for each transcript.

Supplementary Table 2. Genome overlap of *H. glycines* transcripts. SCN transcripts were mapped against the sequenced proteomes from seven nematode species with various feeding behaviors using BLASTX. The transcripts mapping to each proteome were then compared to identify SCN transcripts overlapping with only a single other nematode species and compiled.

Supplementary Table 3. '*Candidatus*' Cardinium hertigii-associated transcripts. Transcripts mapping to the proteome for '*Candidatus*' Cardinium hertigii [27] were identified and available gene ontology (GO) terms were assigned using BLAST2GO.

Supplementary Table 4. Differential expression of known SCN effectors between compatible and incompatible host interactions. Genes were compared between ppJ2 and pJ2 samples in compatible and incompatible conditions. A p-value cutoff of 0.05 and minimum expression change of 2-fold was used to identify differentially expressed genes.

Supplementary Table 5. Effector candidates. List of effector candidate transcripts generated by either the SignalP or N-Preffector based effector prediction pipelines.

Supplementary Table 6. Effector candidates of note. List of novel effector candidates of particular interest due to their annotation and homology to other nematode effectors.
